# The root endophytic bacterial community of *Ricinus communis* L. resembles the seeds community more than the rhizosphere bacteria independent of soil water content

**DOI:** 10.1038/s41598-021-81551-7

**Published:** 2021-01-26

**Authors:** Stephanie E. Hereira-Pacheco, Yendi E. Navarro-Noya, Luc Dendooven

**Affiliations:** 1 Laboratory of Soil Ecology, Cinvestav, Mexico City, Mexico; 2 Cátedras CONACYT, Centro Tlaxcala de Biología de La Conducta, Universidad Autónoma de Tlaxcala, Tlaxcala, Mexico

**Keywords:** Microbiology, Molecular biology, Environmental sciences

## Abstract

Rhizosphere and root endophytic bacteria are crucial for plant development, but the question remains if their composition is similar and how environmental conditions, such as water content, affect their resemblance. *Ricinus communis* L., a highly drought resistant plant, was used to study how varying soil water content affected the bacterial community in uncultivated, non-rhizosphere and rhizosphere soil, and in its roots. Additionally, the bacterial community structure was determined in the seeds of *R. communis* at the onset of the experiment. Plants were cultivated in soil at three different watering regimes, i.e. 50% water holding capacity (WHC) or adjusted to 50% WHC every two weeks or every month. Reducing the soil water content strongly reduced plant and root dry biomass and plant development, but had little effect on the bacterial community structure. The bacterial community structure was affected significantly by cultivation of *R. communis* and showed large variations over time. After 6 months, the root endophytic bacterial community resembled that in the seeds more than in the rhizosphere. It was found that water content had only a limited effect on the bacterial community structure and the different bacterial groups, but *R. communis* affected the bacterial community profoundly.

## Introduction

The rhizosphere can be defined as a complex environment with a wide range of microorganisms that participate in decomposition of organic matter and biogeochemical cycles of N and P, and other plant nutrients^1,2^. Three distinct zones can be distinguished in the rhizosphere, the rhizosphere per se (soil surrounding by roots), the rhizoplane (root surface) and the root itself. The microorganisms in the rhizosphere affect plant development in different ways and are themselves affected by the plant^3^. Most microorganisms in the rhizosphere use plant exudates and dead roots as C substrate thereby competing with the plants for essential nutrients, such as inorganic N and P, while other mineralize soil organic matter releasing plant nutrients. Some microorganisms, such as mycorrhizal fungi, however, help plants to obtain nutrients and stimulate resistance against pathogens^4^. A wide range of bacterial groups generally known as plant growth promoting bacteria (PGPB) facilitate plant nutrient uptake, such as nitrogen, phosphorus and iron. Others modulate plant hormone dynamics by synthesizing one or more phytohormones, such as auxin, cytokinin and gibberellin^5^. As such, the rhizosphere microorganisms interfere directly or indirectly in plant development and the microbial community structure is different from the surrounding soil as a result of the rhizosphere environment^6^.

Endophytic bacteria can be defined as those bacteria that colonize the internal tissue of plants showing no external sign of infection or negative effect on their host and play an important role in plant growth, development and resistance to biotic and abiotic stresses^7,8^. Endophytes enter the plant mainly through the roots that control entry of the soil bacteria from the rhizosphere and rhizoplane, or they enter through aerial parts, such as flowers and stems, and/or were embedded in the seeds^9,10^.

There are two categories of factors that can affect plant growth: abiotic (nutrient availability, temperature, salinity, aeration and soil water content) and biotic (living organisms)^11^. An analysis of the factors that reduced crop yields in the United States over four decades found that drought counted for a 40.8% loss, excess water 16.4% and only 7.2% was due to insects and diseases^12^. Research into the plant response to water stress is important as most climate-change scenarios suggest a decrease in precipitation in many regions of the world that will affect agriculture^13^. These changes in soil water content will not only affect plants, but also the rhizosphere and endophytic microbial communities and how microorganisms interact with the plant and vice versa^14^. *Ricinus communis* L.*,* commonly known as “castor bean” has been cultivated around the world for medicinal use and as an important source of non-edible oil, which has many industrial uses, e.g., in the paint and varnish industry^15^. The plant grows in the tropics and subtropics and is well adapted to a wide range of temperatures and is highly tolerant to biotic and abiotic stresses, such as drought^16^.

The aim of this study was to investigate how cultivation of *R. communis* affected the bacterial community in uncultivated, non-rhizosphere and rhizosphere soil and its roots under different watering regimes. Additionally, the bacterial community in seeds was also determined to investigate how it compared to the endophytes of the roots and the bacteria in the rhizosphere and uncultivated soil. It was hypothesized that the bacterial community would be different in the cultivated and uncultivated soil and that the different watering regimes would affect those differences. Castor bean was used as “model” as it is highly tolerant to drought^16^. Plants were cultivated in the greenhouse under three different watering regimes. In the first treatment, soil was adjusted to 50% water holding capacity (WHC) with distilled water twice a week (considered the wet soil). In the second treatment, soil was adjusted to 50% WHC with distilled water once every two weeks (considered the dry soil) and in the third treatment soil was adjusted to 50% WHC once a month (considered the extreme dry soil). Plant development and the bacterial community structure was determined in the roots, the rhizosphere, the non-rhizosphere soil and uncultivated soil at the onset of the experiment when the different watering regimes were first applied and after 2, 4 and 6 months.

## Results

### Plant growth

Watering regime had a significant effect on all plant characteristics measured after 2, 4 and 6 months (*P* < 0.05) (Table 1). Plant and root dry biomass, and plant growth were reduced strongly in both the dry and extreme dry soil, with a stronger negative effect in the latter than in the first.

**Table 1:** Characteristics of *Ricinus communis* L. plants watered every week (control), once every two weeks (dry) and once a month (extreme dry).

Time (months)	Treatment	Root length (cm)	Length of the above ground part of the plant	Number of leaves	Dry biomass		Fresh biomass	
					Above ground part of the plant	Root (g)	Above ground part of the plant	Root
2	Wet	55.5 A ^a^	78.8 A	10 A	22.7 A	7.9 A	166.2 A	54.7 A
	Dry	44.5 A	66.0 B	8 B	11.4 B	3.6 B	79.8 B	18.1 B
	Extreme dry	38.4 B	43.3 C	5 C	8.2 B	1.9 B	41.4 C	9.4 B
4	Wet	56.8 A	93.0 A	10 A	35.2 A	14.5 A	161.2 A	55.1 A
	Dry	41.1 B	88.7 A	9 A	18.6 B	5.3 B	103.5 B	27.5 B
	Extreme dry	38.7 B	51.2 B	6 B	6.2 C	2.6 B	48.8 C	13.2 C
6	Wet	65.0 A	109.2 A	9 A	53.1 A	17.8 A	176.3 A	73.7 A
	Dry	51.7 B	106.3 A	8 A	30.8 B	5.7 B	107.7 B	25.5 B
	Extreme dry	44.1 B	64.9 B	6 B	13.5 C	3.0 B	65.6 C	16.7 B

Values with the same letter are not significantly different within the watering regimes, i.e. within the columns, after 2, 4 or 6 months (*P* < 0.05). An analysis of variance (aov function in R) was applied and the Tukey post hoc test was used (HSD-test).

### Alpha diversity analysis

A total of 1,779,050 bacterial sequences were obtained from the soil, root and seed samples. A total of 13,653 OTUs at 97% of similarity were clustered after removing singletons, chimeric and organellar sequences from the sequence data. Determining more sequences would have yielded only a limited number of OTUs more, as the rarefication curves were asymptotic (Supplementary Figure S1).

Taxonomic richness and diversity of the soil bacteria was not affected by watering regime and by cultivation of *R. communis* while richness of the endophytic root communities was 25% of that in the rhizosphere (Supplementary Table S1). Taxonomic diversity of soil bacteria was not affected significantly by watering regime or cultivation of *R. communis* considering Shannon and Simpson indexes. Only the Simpson evenness decreased significantly in the wet treatment in the order uncultivated soil > non-rhizosphere soil, rhizosphere > roots, presumably due to the dominance of *Kaistobacter*. Diversity of the endophytic root communities was not affected by watering regime considering the Shannon and Simpson indexes.

The Simpson evenness decreased significantly in the wet treatment in the order uncultivated soil > non-rhizosphere soil, rhizosphere > roots (Supplementary Table S1). The Simpson evenness decreased in the order uncultivated soil > rhizosphere > non-rhizosphere > roots after 6 months. Richness and diversity of endophytic root communities decreased sharply with time. Bacterial richness decreased 68% after 6 months and the Simpson evenness decreased from 0.147 to 0.019 due to the dominance of *Pseudomonas*.

### The effect of cultivation of *Ricinus communis* on the bacterial community structure

**Figure 1:**
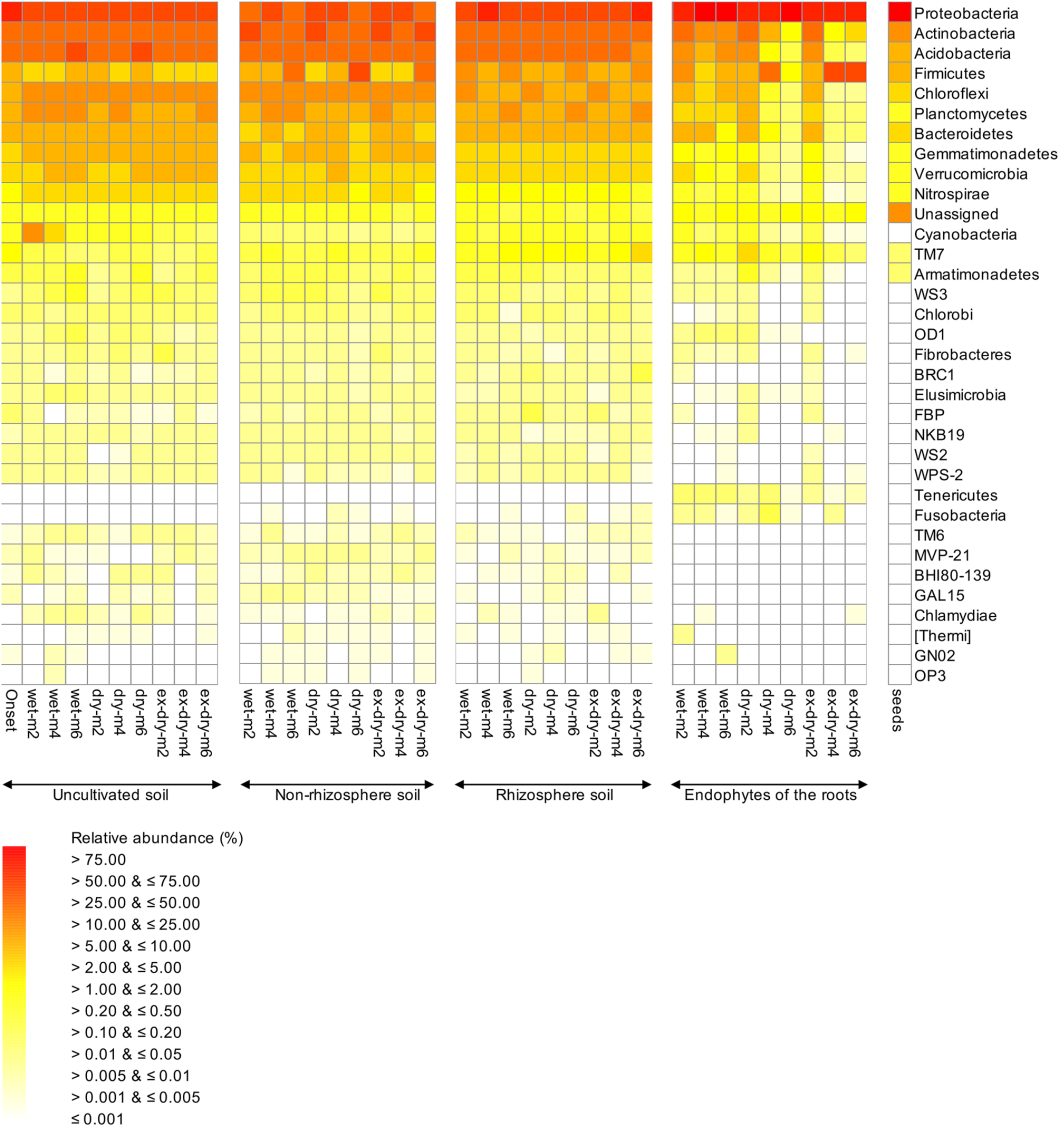
Heat map with the relative abundances of the bacterial phyla in the wet, dry and extreme dry (ex-dry) uncultivated, non-rhizosphere and rhizosphere soil, and the endophytes of the roots and seeds of *Ricinus communis* L after 2 (m2), 4 (m4) and 6 months (m6). Wet soil was adjusted to 50% water holding capacity (WHC) with distilled water twice a week, dry soil once every two weeks and extreme dry soil once a month.

**Figure 2:**
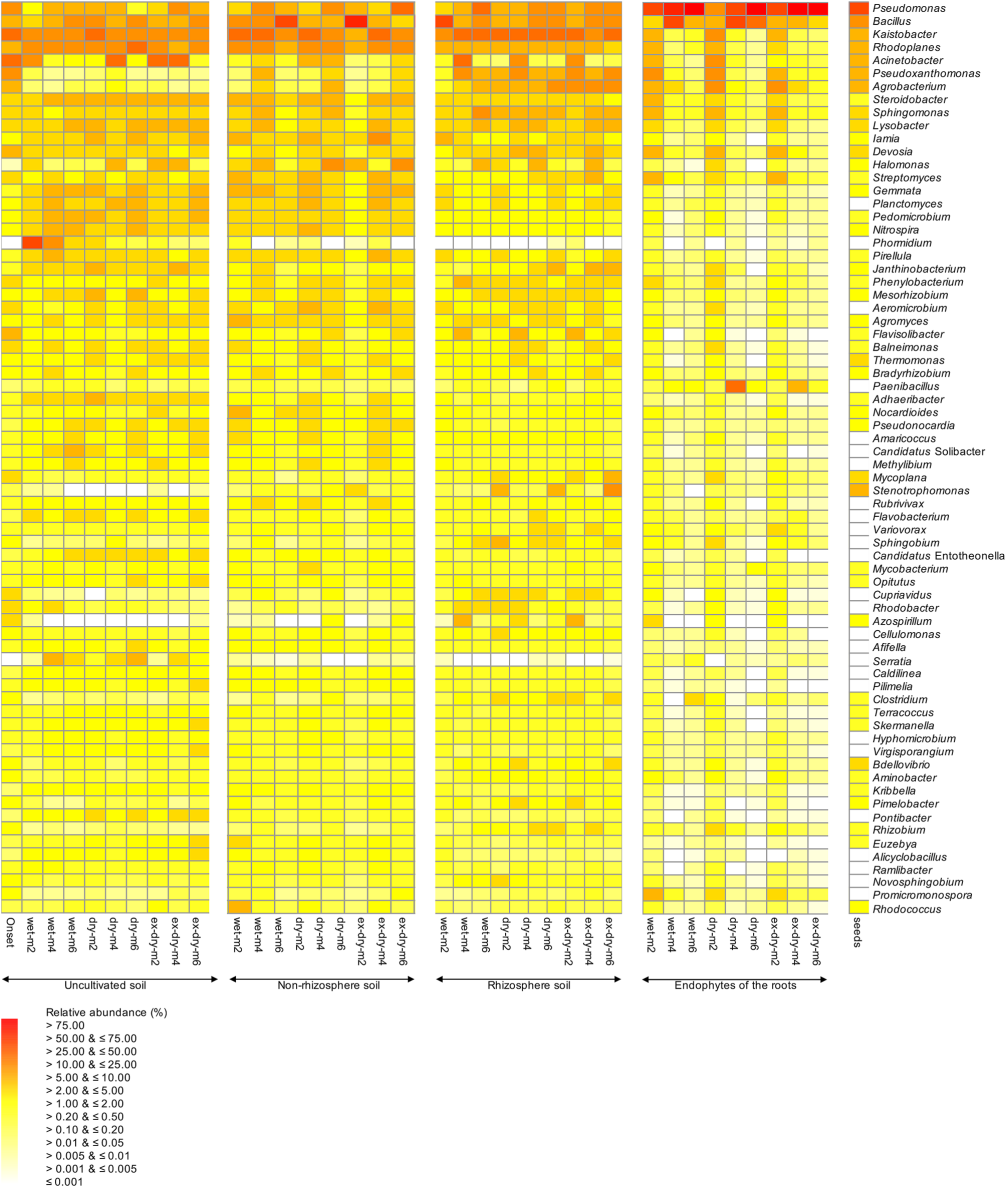
Heat map with the relative abundances of the 70 most abundant bacterial genera in the wet, dry and extreme dry (ex-dry) uncultivated, non-rhizosphere and rhizosphere soil, and the endophytes of the roots and seeds of *Ricinus communis* L after 2 (m2), 4 (m4) and 6 months (m6). Wet soil was adjusted to 50% water holding capacity (WHC) with distilled water twice a week, dry soil once every two weeks and extreme dry soil once a month.

At the onset of the experiment, the soil was dominated by Proteobacteria (relative abundance 51.61 ± 3.42%), Actinobacteria (17.78 ± 0.80%) and Acidobacteria (10.18 ± 1.39%) (Fig. 1). The most dominant genera were *Kaistobacter* (11.84 ± 2.37%), *Acinetobacter* (9.55 ± 5.66%) and *Pseudoxanthomonas* (8.00 ± 0.04%) at the onset of the experiment (Fig. 2). Cultivation of *R. communis* had a highly significant effect on a wide range of bacterial groups independent of the watering regime as obtained with an analysis of differential abundance (ALDEx2) (*P* < 0.0001) (Supplementary Figure S2, S3, Table S2). For instance, 35 of the 312 bacterial groups assigned to the level of genus and 6 of the 35 phyla were highly significantly affected by cultivation of *R. communis* (*P* < 0.001) and a total of 95 genera and 16 phyla were affected significantly (*P* < 0.05). Considering the most abundant bacterial phyla, the relative abundance of OTUs belonging to the Firmicutes was higher in the non-rhizosphere compared to the uncultivated soil independently of watering regime applied to soil and that of Firmicutes and TM7 in the rhizosphere (Figure S2). The relative abundance of Firmicutes, Proteobacteria and TM7 was larger in the roots than in the uncultivated soil independently of watering regime applied to soil, while it was lower for most other bacterial phyla. The relative abundance of OTUs belonging to *Agrobacterium* and *Pseudoxanthomonas* was higher in the rhizosphere compared to the uncultivated soil independently of watering regime applied to soil (Figure S3). The relative abundance of most bacterial genera decreased in the roots of *R. communis* compared to the uncultivated soil and only members of *Pseudomonas* were enriched.

**Figure 3:**
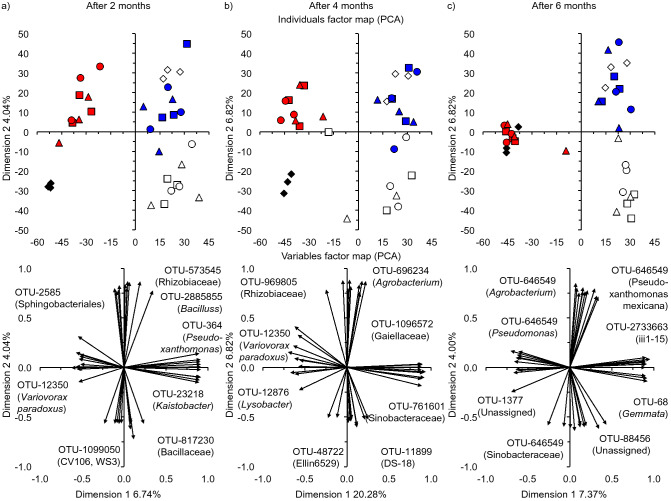
A principal component analysis (PCA) with the converted sequence counts of all operational taxonomic units (OTUs) using the centred log-ratio transformation, i.e. aldex.clr argument (ALDEx2 package^75^) after (**a**) 2 months, (**b**) 4 months and (**c**) 6 months of applying the different watering regimes in the soil at the onset of the experiment (open triangle), the rhizosphere in the wet soil (Blue square), the dry soil (blue circle) and the extreme dry soil (blue triangle), the uncultivated wet soil (open square), the dry soil (open circle) and the extreme dry soil (open triangle), the endophytic bacteria in the roots of *Ricinus communis* L. in the wet soil (red square), the dry soil (red circle) and the extreme dry soil (red triangle), and the seeds (red diamond).Wet soil was adjusted to 50% water holding capacity (WHC) with distilled water twice a week, dry soil once every two weeks and extreme dry soil once a month.

A PCA was used to represent a multivariate data table as smaller set of variables so as to determine how the soil bacterial communities were affected by cultivation of *R. communis,* and how they compared to the endophytic root and seed bacterial communities. A PCA with the bacterial groups up to the level of genus and all the OTUs in the uncultivated soil, the rhizosphere soil and the endophytic root community after 2 or 4 months, and that in the seeds and in the soil at the onset of the experiment visualized four groups (Fig. 3a, b, Supplementary Fig. 4a, b). A first group contained the bacterial community in the uncultivated soil, a second the rhizosphere soil and the uncultivated soil at the onset of the experiment, a third the endophytic root bacteria and a fourth the bacterial community in the seeds. A PCA with the bacterial groups up to the level of genus and all OTUs in the uncultivated soil, the rhizosphere soil and the endophytic root community after 6 months, and that in the seeds and in the soil at the onset of the experiment indicated that the bacterial community in the seeds resembled that in the roots more than in the rhizosphere or the uncultivated soil (Fig. 3c, Supplementary Fig. 4c). The bacterial communities in the uncultivated and rhizosphere soil and the roots were similar in the wet, dry and extreme dry soil independently of the taxonomic level considered after 2, 4 and 6 months (Fig. 3, Supplementary Figure S4). A cluster analysis confirmed the separation between the soil samples as visualized with the PCA. The rhizosphere soil was grouped with the uncultivated soil at day 0, while the bacterial community structure in the roots resembled that in the seeds (Supplementary Figure S5). The bacterial community in the uncultivated soil after 2, 4 and 6 months was most separated from the others. Consequently, cultivation of *R. communis* had a highly significant effect on the bacterial community structure considering the different bacterial taxonomic levels (*P* < 0.001), but not the time of sampling and the interaction between the time of sampling and cultivation of *R. communis* (Table 2). Pairwise perMANOVAs with all OTUs using the sequence counts converted with the centred log-ratio transformation showed that the bacterial community in the uncultivated soil, non-rhizosphere soil, rhizosphere soil and the endophytic root community were highly significant different between them (Supplementary Table S3).

**Table 2:** Effect of cultivation of *Ricinus communis* L. (uncultivated, non-rhizosphere and rhizosphere soil, and roots) and time (2, 4 or 6 months) on bacterial community composition at different taxonomic levels using a compositional approach ^a^.

Factor	Wet soil		Dry soil		Extreme dry soil	
	F value	*P* value	F value	*P* value	F value	*P* value
**Phyla**						
Cultivation	5.92	** < 0.001** ^b^	7.23	** < 0.001**	9.08	** < 0.001**
Time	1.21	0.221	1.18	0.255	1.28	0.213
Cultivation*Time	1.54	0.074	1.61	0.083	1.61	0.052
**All taxonomic groups assigned to the level of bacterial genus**						
Cultivation	2.99	** < 0.001**	3.47	** < 0.001**	3.64	** < 0.001**
Time	1.24	0.114	1.31	0.104	1.33	0.104
Cultivation*Time	0.99	0.459	1.15	0.169	1.10	0.209
**All operational taxonomic units (OTUs)**						
Cultivation	1.65	** < 0.001**	1.66	** < 0.001**	1.74	** < 0.001**
Time	0.98	0.529	1.03	0.230	1.02	0.278
Cultivation*Time	1.00	0.444	0.99	0.528	1.02	0.303

^a^ perMANOVA analysis with sequence counts converted using the centred log-ratio transformation, i.e. aldex.clr argument (ALDEx2 package^75^) (aldex.clr(stephcounts, mc.samples = 128, denom = "all", verbose = FALSE, useMC = FALSE)). The adonis2 argument (Vegan package, Oksanen et al., 2019) was used for the perMANOVA analysis (#adonis2(stephclr ~ culivation*time, data = stephcode, permutations = 999, method = "euclidean"), ^b^ *P* values in bold indicate a significant effect.

### The effect of the different watering regimes on the bacterial community structure

None of the bacterial genera or phyla was affected significantly by the different watering regimes applied to the non-rhizosphere soil and only a limited number was affected significantly in the uncultivated and rhizosphere soil, and in the roots (Supplementary Table S4, Supplementary Figure S6). The PCA did not separate the bacterial community in the wet, dry or extreme dry soil in the uncultivated, non-rhizosphere and rhizosphere soil or in the roots independent from the bacterial taxonomic level considered (Supplementary Figure S7). However, the bacterial community structure showed shift in time most accentuated in the roots. Consequently, the perMANOVA analysis showed no significant effect of watering regime on the bacterial community structure independent of the taxonomic level considered, but there was a significant effect of time in the non-rhizosphere and rhizosphere soil and in the roots (Table 3).

**Table 3:** Effect of water regime (WC, wet, dry and extreme dry soil), time (2, 4 or 6 months) and their interaction on bacterial community using a compositional approach ^a^.

Factor	Uncultivated			Non-rhizosphere		Rhizosphere		Endophytic Root	
	F value	*P* value		F value	*P* value	F value	*P* value	F value	*P* value
**Phyla**									
WC	1.29	0.113	0.83		0.715	1.32	0.110	1.38	0.089
Time	0.66	0.871	1.41		0.145	1.20	0.248	2.35	**0.002** ^b^
WC*Time	0.91	0.634	0.44		0.996	1.05	0.374	1.00	0.476
**All bacterial groups assigned to the level of genus**									
WC	0.96	0.767	0.93		0.861	0.98	0.628	1.18	0.065
Time	0.99	0.528	1.43		** < 0.001**	1.37	** < 0.001**	2.16	** < 0.001**
WC*Time	0.92	0.941	0.88		0.985	0.98	0.610	1.01	0.409
**All operational taxonomic units (OTUs)**									
WC	0.99	0.697	0.97		0.880	1.01	0.231	1.02	0.162
Time	0.98	0.729	1.09		**0.033**	1.08	**0.005**	1.14	** < 0.001**
WC*Time	0.96	0.986	0.93		0.999	1.00	0.587	1.00	0.399

^a^ perMANOVA analysis with sequence counts converted using the centred log-ratio transformation, i.e. aldex.clr argument (ALDEx2 package^75^) (aldex.clr(stephcounts, mc.samples = 1000, denom = "all", verbose = FALSE, useMC = FALSE)). The adonis2 argument (Vegan package^74^) was used for the perMANOVA analysis (#adonis2(stephclr ~ loc*ti, data = stephcode, permutations = 999, method = "euclidean"), ^b^ *P* values in bold indicate a significant effect.

## Discussion

The study of how plants respond to prolonged drought stress is becoming more important as climate change will induce more erratic precipitation in many parts of the world^17^. The most typical effect of drought on plants is a reduction in growth due to changes in ion balances, water status, photosynthetic efficiency, and carbon allocation and utilization^18^. Other changes occur also, such as a reduction in leaf area, early maturity and prolonged stomata closure to mitigate the possible effects of drought^19,20^. The number of leaves decreases, as found in this study, as the plant shed its older leaves earlier, while the remaining ones have often a higher photosynthetic rate^21^. The metabolism and concentration of sucrose, the major form of carbon transportation in plants, is reduced under osmotic stress as a result of changes in the photosynthesis process. Drought leads to a reduction in root and shoot dry weight as found in this study. However, root length was more affected by drought than the shoot length as observed in other studies with castor bean^18,22^.

In general, the cultivation of *R. communis* and the watering regimes did not have an effect on the soil bacterial richness and diversity. Likewise, the rhizosphere of rice (*Oryza sativa* L.), barley (*Hordeum vulgare* L.) and *Arabidopsis* plants exhibited a similar diversity as compared to uncultivated soil^23–25^⁠⁠. Tóth et al.^26^ found no significant difference in alpha diversity between the control and drought-treated soil, while Armstrong et al.^27^ reported little or no temporal variation in alpha diversity in a hot desert soil. Richness and diversity of soil bacterial communities in the rhizosphere or in the uncultivated soil did not change over time.

The endophytic bacterial communities of plants are generally less rich and diverse than in the rhizosphere as endophytes first must interact successfully with the plant to survive and then reduce the entrance of other microorganisms through the stimulation of plant defense^28^. Bacterial richness in the roots of *R. communis* represented 25% the bacterial richness in the rhizosphere. Similar results have been reported for different plants, e.g., a large number of grasses (Poaceae)^29^, tomato plants^30^ (*Solanum lycopersicum* L*.*)^23^, rice and *Arabidopsis thaliana*^25^. In this study, *R. communis* restricted even more the bacteria in its roots over time, retaining mostly *Pseudomonas* OTUs. Although a variety of microbes may enter and become transient endophytes they are subsequently filtered through different mechanisms, e.g. interactions with the host, niche utilization attributes, and competition with established endophytes^28^. *Pseudomonas* fulfills these characteristics as it interacts positively with a variety of plant host, possesses plant growth-promoting characteristics, such as nitrogen fixation and production of plant hormones or antimicrobial substances, and induce systemic plant defense response^31^.

The rhizosphere environment is different from the surrounding soil as more easily decomposable organic material, mostly root exudates and dying roots^32^, is available for heterotrophs and microorganisms must compete with plants for nutrients and water. The diversity, amount and activity of microorganisms in the rhizosphere are determined by the release of root exudates, which differ between plant species and enriches microorganisms with different functions or abilities. Some competition exists between plants and microorganisms, but some bacteria are beneficial. The fact that plants reshape the microbiome by selecting microorganisms with beneficial functions leads to mutualistic relationships in the rhizosphere^33^. Consequently, some bacterial groups will be enriched while the relative abundance of others will decrease.Dai et al.^34^ reported that the abundance of Actinobacteria and Acidobacteria increased in the rhizosphere of peanut (*Arachis hypogaea* L.) plants during the seedling and podding stages in drought-treated soil, while that of Cyanobacteria and Gemmatimonadetes increased in the flowering stage. Xu et al.^35^ reported that Firmicutes were enriched in the rhizosphere soil of sorghum and Acidobacteria depleted under dry conditions. In this study, the relative abundance of Acidobacteria, Actinobacteria and Gemmatimonadetes decreased in the rhizosphere compared to the uncultivated soil independent of the soil water content, while that of Cyanobacteria increased. The relative abundance of Firmicutes increased in the non-rhizosphere soil but showed no clear pattern in the rhizosphere. As such, soil properties, and plant characteristics and development stages define how bacteria will be affected by the rhizosphere^25,36^.

Some bacterial genera, all belonging to the Proteobacteria, were enriched highly in the rhizosphere of *R. communis* compared to the uncultivated soil e.g., *Pseudoxanthomonas*, *Rhizobium* and *Acidovorax*. These genera are often enriched in the rhizosphere. For instance, *Pseudoxanthomonas* and *Acidovorax* in the rhizosphere of maize (*Zea mays* L.)^37^, and members of *Rhizobium* were ubiquitous in the rhizosphere of legumes^38^.

The non-rhizosphere soil was affected by the cultivated plant, but not to the same extent as the rhizosphere. Small roots can be part of the non-rhizosphere soil in this experiment, so the organic C content is higher than in the uncultivated soil, but lower than in the rhizosphere. Additionally, conditions in the non-rhizosphere soil are also different from the rhizosphere as competition for nutrients and water is less accentuated^39^.

Copiotrophs should be enriched in the non-rhizosphere and rhizosphere soil compared to the uncultivated soil, while the relative abundance of oligotrophs should decrease. Actinobacteria and Firmicutes are widely recognized as copiotrophs^40,41^ so they should be enriched in the non-rhizosphere soil and rhizosphere compared to the uncultivated soil, while the relative abundance of Acidobacteria, Bacteroidetes and Verrucomicrobia, considered oligotrophs, should decrease^42–44^. Some bacterial groups such as Acidobacteria and Verrucomicrobia behaved clearly as they have been described, i.e., oligotrophic, in both the rhizosphere and non-rhizosphere soil, but others not. Actinobacteria were enriched mostly in the non-rhizosphere soil, but depleted in the rhizosphere, while Bacteroidetes were enriched mostly in both environments although they have been described as oligotrophs. Firmicutes were enriched in the non-rhizosphere soil and after 2 months in the rhizosphere, but their relative abundance decreased sharply after 6 months in the rhizosphere. This confirms again that plant type and development, and distance from the plant roots, and soil characteristics all affect bacterial groups in the non-rhizosphere and rhizosphere soil^25,36,45^.

Edwards et al.^23^ reported that Proteobacteria, Firmicutes, Actinobacteria and Acidobacteria were enriched in the endophytic community of rice. In this study, only OTUs belonging to the Proteobacteria were always enriched in the roots of *R. communis* independent of time of sampling or soil water content. Xu et al.^35^ reported that during the early development of the root microbiome of sorghum the bacterial community is dynamic, with large shifts in many dominant taxa, e.g., Actinobacteria, Firmicutes, and Proteobacteria. In this study, large changes in the relative abundance of Actinobacteria and Firmicutes were detected over time, but variation in that of Proteobacteria was smaller. Members of *Bacillus* and *Pseudomonas* were enriched in sweet potato (*Ipomoea batatas* L. Lam.) tuberous roots^46^ and tree type of peonies^37^. In this study, members of *Pseudomonas* were always enriched in the endophytic root community of *R. communis* and OTUs belonging to *Paenibacillus* nearly always.

In the uncultivated soil, the easily decomposable organic material will be mineralized and depleted quickly as it is not replenished. The depletion of the easily decomposable organic material will alter the bacterial community structure. The conditions and characteristics of the plant rhizosphere are different from an uncultivated soil, which have a profound effect on the bacterial community structure^47^. In the rhizosphere, plant exudates provide heterotrophs with an easily decomposable C substrate, but they compete with the soil microorganisms for nutrients, e.g., nitrogen and phosphorus^48^. The root microbiome can be considered a sub-set of the rhizosphere microorganisms. Its composition will be defined by plant type and development stages, and environmental conditions although the bacteria in the seeds might play a role too^25,36^.

Already after 2 months in this study, the bacterial community structure in the uncultivated soil was different from that at the onset of the experiment and different from that in the rhizosphere and the roots. Liberation of root exudates supplied the bacteria in the rhizosphere with organic material. As such, the bacterial community structure in the rhizosphere was similar as found at the onset of the experiment. The endophytic root bacterial community was distinct from that in the rhizosphere indicating that there was a clear barrier between “the inner part” and the “outer part” of the plant. Interestingly, the root bacterial community structure after 6 months resembled that in the seeds and not in the rhizosphere. This would suggest that the barrier between the rhizosphere and the inner roots was stronger than between the different plant parts, e.g. roots and seeds. As such, *Ricinus* selects its hosts and controls its microbiome. Initially, a subset of microorganisms is enriched in the rhizosphere and a subset of these microorganisms colonize the roots. This colonization of the roots and subsequently the whole plant is affected by different factors, such as the different plant development stages, and related presumably to specific functions of the endophytes^49^. The effect of plant development on microbial populations might occur in the rhizosphere and endosphere, and be related to a defense against pathogens and/or nutrient uptake, such as N. Nitrogen is required in higher concentrations in later of stages of plant development^50^. So it can be hypothesized that in early stages, plants release a high diversity and amount of substances as substrates for a wide range of microorganisms, but as the plant ages it releases specific substrates in an effort to select a particular subset of microbes^50^.

This would also suggest that plants enrich bacterial groups in their roots that are not necessarily the most abundant in the rhizosphere or surrounding soil. This might also explain why fertilizing plants with microorganisms to enrich their biome and to stimulate their growth might have only a temporary effect on plant development^51^. However, caution should be taken, and further research will be necessary to confirm these results as different techniques were used to extract DNA from the seeds and roots, and from the soil, which might have biased the results obtained.

In the uncultivated wet soil, the organic material was mineralized and depleted quickly as environmental conditions were favourable for bacterial metabolic activity, e.g., a large water availability. The depletion of the easily decomposable organic material will alter the bacterial community structure. In the dry or extreme dry uncultivated soil, changes in the bacterial community will not only be controlled by a reduction of the easily available organic material, but also by the fluctuating water content. When a soil dries out, microbial activity will decrease^52^ and microorganisms that can cope with the drying-rewetting cycle will be enriched. A subsequent rewetting will trigger a cascade of responses increasing metabolic and enzymatic activities thereby enriching some bacterial groups while depleting others^53^. Soil properties might also affect how the watering regime affects the bacterial communities. For instance, aggregates control the connectivity between microorganisms in soil by isolating the intra-aggregate communities from one another during dry periods and allowing the transportation of solutes, metabolites and genetic material, when the soil is wet. When the soil is dry, each aggregate community may function within its local environment independently from other communities and may transfer new functional capacities when soluble carbon is released upon wetting^54^⁠.

In this study, however, the microbial community structure was not affected by water content in any soil sample or root-associated sample. As such, soil characteristics, length and severity of the drought will determine if the bacterial community structure was affected. Members of Actinobacteria have been found to be resistant to drought^14,55^. This could be due to their filamentous growth and ability to form spore, which facilitate survival in dry conditions. Additionally, Actinobacteria are Gram-positive bacteria and they are characterized by a thicker peptidoglycan cell wall, which can make them more resistant to drought^56^. This was confirmed in the uncultivated soil of this study as their relative abundance was higher in the dry soil and extreme dry soil compared to the wet soil.

In the rhizosphere, plant exudates provide heterotrophs with an easily decomposable C substrate and although some microorganisms assist the plant in the uptake of nutrients, e.g., nitrogen and phosphorus, others will compete with the plant. As such, the effect of watering regime or time of sampling might be different from that in the uncultivated or non-rhizosphere soil^48^. Extreme drought might also induce leaking of cellular nutrients from the roots, which might alter the bacterial community structure even further^57^. Santos-Medellín et al.^58^ found changes in the relative abundance of bacterial communities in the rice rhizosphere after drought, but Hartmann et al*.*^55^ reported no changes in the microbial community structure. In this study, different watering regimes had no significant effect on the bacterial community structure in the rhizosphere.

Dai et al.^34^ reported that the abundance of Actinobacteria and Acidobacteria increased in the rhizosphere of peanut (*Arachis hypogaea* L.) plants during the seedling and podding stages in drought-treated soil, while that of Cyanobacteria and Gemmatimonadetes increased in the flowering stage. They suggested that not only drought, but also the developmental stages of the peanut plant affected the structure of the microbial community. In this study, only a limited number of genera were affected always in the same way in the rhizosphere. The relative abundance of genera always increased (e.g., *Agrobacterium*, *Janthinobacterium* and *Promicromonospora*) or decreased (e.g., *Novosphingobium*) in the dry and extreme dry soil with time. As a result, the bacterial community in the rhizosphere changed highly significantly over time considering the bacterial genera suggesting an effect of plant development on them.

The root-associated microbiome is influenced by many biotic and abiotic factors^23,25,36^. Water availability, however, had no significant effect on the endophytic bacterial community in this study. Santos-Medellín et al.^58^ reported that drought significantly changed the overall bacterial composition in the rhizosphere and the endosphere of rice plants and unplanted soils, with the largest changes found in the rhizosphere and endosphere. They also found that the response of the bacterial groups to drought was consistent taxonomically in different soils and cultivars and was primarily driven by an enrichment of multiple Actinobacteria and Chloroflexi, as well as a depletion of several Acidobacteria and Deltaproteobacteria*.* In this study, the different watering regimes had only a limited effect on the bacterial community structure. The response of bacterial groups to the different watering regimes, however, changed often with time. These changes over time might be due to soil type, experimental conditions and type of plant^23,36,45^.

Xi et al.^59^ found a strong relationship between the cell wall structure in bacterial groups and resistance to drought. In this study, no such clear relationship was found. For instance, Xi et al.^59^ stated that *Actinoplanes*, a member of monoderm Actinobacteria normally enriched in drought, was less resistant to drought as it has a different type of cell wall than other members of the Actinobacteria. However, in this study, Actinobacteria were enriched in the uncultivated dry and extreme dry soil compared to the wet soil, but their relative abundance decreased in the roots under dry conditions while their response to watering regime was variable in the rhizosphere and non-rhizosphere soil (Supplementary Table S5).

## Conclusion

The bacterial community structure was affected significantly by the presence of *R. communis* and the watering regime had only a limited effect on the bacterial community structure in the uncultivated, non-rhizosphere and rhizosphere soil, and roots. This showed that availability and composition of organic material, in this study trumped watering regime in its effect on the microbial community.

The bacterial community in the rhizosphere was similar to that at the onset of the experiment independent of the watering regime, but different from that in the uncultivated soil and the roots. The endophytic bacterial root community resembled that in the seeds. As such, *R. communis* exercised a strong effect on its root biome, but the composition of the initial bacterial community and the changes in the soil water content had a much smaller effect on the root biome.

Although water content had sometimes an effect on a specific bacterial group in the uncultivated, non-rhizosphere or rhizosphere soil, or the roots of *R. communis*, the effect changed with watering regime and time of sampling. Cell wall structure, which could be considered a major indicator of drought resistance, could not explain the effect of water content on bacterial groups. As such, a combination of the initial bacterial community structure, organic material availability and composition, the presence of roots, plant type and development stage, and soil characteristics defined how bacterial groups responded to drought. Pinpointing one factor that controls the bacterial community in different ecosystems might be difficult or impossible as a combination of the factors above mentioned will determine a possible effect.

## Methods

### Sampling experimental site and chemical analysis

Soil was collected at the “*Centro de Investigación y Estudios avanzados del Instituto Politécnico Nacional”*, Mexico City (lat. 19°30′ 28.7ʺ N; 99°07′ 50.8ʺ W, altitude 2240 m above sea level) on 25 of February 2016. Three different 400 m^2^ areas were defined and the 0–20 cm top soil layer collected. Approximately, 300 kg soil was collected from each area, passed separately through a 0.5 cm sieve so that three soil samples (*n* = 3) were obtained. The field-based replication (*n* = 3) was maintained in the laboratory experiment so as to avoid pseudo-replication. The sandy loam soil had an organic C content of 36.66 g C kg^−1^ soil and a total N content of 1.96 g N kg^−1^ soil. The WHC was 584 g kg^−1^ soil, electrolytic conductivity (EC) 1.92 dS m^−1^ and pH 7.8. Details of how the soil characteristics were determined can be found in Patiño-Zúñiga et al.^60^.

### Seed collection, plant growth conditions and treatments

On the 1st of March 2016, seeds were collected from one castor bean plant (one phenotype) along the edge of the Rio de los Remedios in Mexico City (México) (Supplementary Figure S8). Homogeneous seeds were surface sterilized with 1% sodium hypochlorite for 15 min and rinsed with distilled water to remove excess solution^61^. The seeds were placed in wet vermiculite and germinated for a month. On the same day, 18 sub-samples of 8 kg soil from each plot (*n* = 3) were separately added to planting pots (570 cm^3^) that contain a 3 cm layer of tezontle, which is a porous, highly oxidized, reddish volcanic rock. The soil was maintained at 50% WHC by adding distilled water every 3 or 4 days.

On 8th of April 2016, a germinated plantlet that had developed a pair of cotyledons, a pair of primary leaves and one or two fully developed main leaves was planted in half of the pots (*n* = 12) while the other half of the pots remained unplanted and was considered the uncultivated soil (Supplementary Figure S8). The pots were placed in the greenhouse with a temperature that ranged between 16 and 35 °C and relative humidity between 20 and 60% for 28 days.

On the 8th of May 2016, a pot with uncultivated soil or a plant was selected at random from each plot (*n* = 3) (Supplementary Figure S8). A 20 g sub-sample of uncultivated soil was collected and extracted for DNA as described below. A pot with a plantlet from each plot (*n* = 3) was taken and the non-rhizosphere and rhizosphere soil was collected. The roots were shaken gently first and the soil that remained attached firmly to the roots was removed by carefully washing with distilled water and considered the rhizosphere soil. The soil that was shaken loose from the roots was collected and considered the non-rhizosphere soil. The non-rhizosphere and rhizosphere soils were stored at − 20 °C until extracted for DNA and analysis as described below.

On the same day, three treatments were applied to both the unplanted and the planted pots (Supplementary Figure S8). In a first treatment soil was adjusted to 50% water holding capacity (WHC) with distilled water twice a week and considered the wet soil (Supplementary Figure S9). In a second treatment soil was adjusted to 50% WHC with distilled water once every two weeks (considered the dry soil) and in a third treatment soil was adjusted to 50% WHC with distilled water once a month (considered the extreme dry soil). The plots were arranged randomly in the greenhouse and kept for 6 months.

After 2 months (8th July 2016), 4 months (6th September 2016) and 6 months (6th November 2016) one plant was removed from each replicate plot (*n* = 3) and each treatment (*n* = 3) (Supplementary Figure S8). After 2 months plants were in the juvenile vegetative stage, after 4 months in the adult vegetative phase, and after 6 months in the adult vegetative stage and starting to bud. Fresh and dry weight of the root and above ground part of the plant biomass, the number of leaves and the length of the roots and above ground part of the plant were determined. The non-rhizosphere and rhizosphere soil and roots were extracted for DNA as described below. Additionally, soil from one pot without plants (uncultivated soil) from each replicate plot (*n* = 3) and treatment (*n* = 3) was removed and a sub-sample was extracted for DNA.

### DNA extraction of samples and PCR amplifications

Soil DNA was extracted with three different methods. A first technique consisted in a chemical lysis, a second in an enzymatic lysis and a third in a lysis by thermal shock^62–64^. A 0.5 g subsample of soil was used for each extraction method in triplicate and the DNA extracted by all three methods was pooled. As such, 4.5 g soil of non-rhizosphere, rhizosphere and uncultivated soil of each treatment (*n* = 3) and plot (*n* = 3) was extracted for DNA.

Collected roots and seeds were washed with sterile water, soaked in ethanol for 5 min and disinfected with 2.5% sodium hypochlorite for 15 min and rinsed with sterile water^65^. The root (1.5 g from each sampled plant) and seeds (1.5 g in triplicate) were macerated with liquid nitrogen to a fine powder. Root DNA was extracted with the enzymatic lysis technique of Sambrook and Russell^63^. The extraction of DNA from the seeds was based mainly on a protocol proposed by CIMMYT^66^, but with some adaptations based on the method described by Pineda et al.^67^. First, tissues were disinfected and then macerated with liquid nitrogen to obtain a fine powder. Nine mL of extraction solution (Tris 1 M, EDTA 0.5 M, CTAB, NaCl 5 M, BME 14 M) was added and incubated at 70 °C for 1 h^66^. Proteins were precipitated with a solution of chloroform/isoamyl alcohol (24:1)^67^. Phenol was used for a higher purity extraction followed by precipitation with ethanol and washed to purify DNA^66^.

Amplicon libraries of V3-V4 regions of 16S rRNA genes were obtained using the primers [341-F (5´-CCTACGGGNGGCWGCAG-3´) and 805-R (5´-GACTACHVGGGTATCTAATCC-3´)] and containing the adapters for Illumina chemistry. The following thermal cycling scheme was used for amplification: initial denaturation at 95 °C for 10 min, 25 cycles of denaturation at 95 °C for 40 s, annealing at 53 °C for 45 s, and extension at 72 °C for 1 min followed by a final extension period at 72 °C for 10 min. All samples were amplified five times, pooled, purified and quantified on a spectrophotometer NanoDrop TM 2000 (Thermo Fisher Scientific Inc., Suwanee, GA). The purified DNA samples were sent to Macrogen, Inc. (DNA Sequencing Service, Seoul, Korea) for 300-pb paired-end (PE) MiSeq runs (Illumina).

### Sequence processing and alpha diversity analysis

The QIIME software (version 1.9.1) was used to analyze the DNA sequences. Paired-end sequences were assembled with fastq-join method within QIIME. Quality filtering was done and sequences with ambiguous base calls and quality values less than 23 Phred Q score were eliminated. Operational taxonomic units (OTUs) were determined at 97% similarity using the UCLUST algorithm with the open reference OTU picking strategy and against the Greengenes 16S rRNA database. Taxonomic assignment was done using the Ribosomal Database Project’s classifier (rdp) against the Greengenes 16S rRNA database with a confidence of 0.8. Sequences were aligned against the Greengenes reference database using PyNAST version 1.2.2^68^. Chimeric sequences, identified using usearch61, and OTUs representing chloroplast or mitochondrial DNA and with less than two observations were eliminated^69^. Alpha diversity indices from root and seed samples were calculated from 3500 rarefied sequences while soil samples were rarefied to 10,000 sequences. The Goods’ coverage at this sequencing depth was on average 88% for root and seeds, and 87% for soil samples (Data not shown). Sequences from the roots were also rarefied at 2000 using the collapse_samples.py function of QIIME^70^.

### Statistical analyses

All statistical analyses were done in R^71^. An ANOVA test (aov function) was used to determine the effect of soil water content on plant characteristics and alpha diversity indexes. Heatmaps of the relative abundances of the bacterial groups were constructed with the pheatmap package^72^. Ordination (principal component analysis (PCA)), multivariate comparison (perMANOVA) and differential abundance (ALDEx2) was done with converted sequence data using the centred log-ratio transformation test returned by the aldex.clr argument (ALDEx2 package^73^). The PCA was done with the vegan package^74^. Effect of water content (wet, dry and extreme dry) or location (uncultivated, non-rhizosphere, rhizosphere and roots) on the bacterial groups was done using a compositional approach (analysis of differential abundance taking sample variation into account, aldex.kw argument, ALDEx2 package^75^). A permutational multivariate analysis of variance (perMANOVA) analysis was also done with sequence counts converted using the centred log-ratio transformation (aldex.clr argument, ALDEx2 package^75^). The adonis2 argument (Vegan package^74^) was used for the perMANOVA analysis (#adonis2(stephclr ~ cultivation of *R. communis* (or different watering regimes)*time, data = stephcode, permutations = 999, method = "euclidean"). Additionally, pairwise perMANOVAs were done with all OTUs using the sequence counts converted with the centred log-ratio transformation with 1000 permutations and using the pairwise.perm.manova function in the RVAideMemoire package^76^, which performs pairwise comparisons between group levels (the effect of cultivation in the wet, dry and extreme dry soil) with corrections for multiple testing using the Benjamini Hochberg adjustment of *P*-values.

The unsupervised cluster analysis was done with the log-ratio-transformed data and with the Aitchison distance metric that fulfills the criteria required for compositional data.

The analysis was done with the "hclust" function of Rstats using the ward.D2 method, that clusters groups together by their squared distance from the geometric mean distance of the group^77^.

The effect of the watering regime on the bacterial groups was determined separately in the uncultivated, non-rhizosphere and rhizosphere soil and in the roots by a ratio in two different ways.

1. when the relative abundance of the bacterial group (mean of values obtained after 2, 4 and 6 months) was higher in the wet soil than in the dry or extreme dry soil then the ratio was calculated as:
  Ratio = (relative abundance of the bacterial group in the wet soil – relative abundance of the bacterial group in the dry or extreme dry soil)/(relative abundance of the bacterial group in the dry or extreme dry soil),
2. when the relative abundance of the bacterial group (mean of values obtained after 2, 4 and 6 months) was lower in the wet soil than in the dry or extreme dry soil then the ratio was calculated as:

Ratio = − (relative abundance of the bacterial group in the dry or extreme dry soil − relative abundance of the bacterial group in the wet soil)/(relative abundance of the bacterial group in the wet soil).

The effect of cultivating *R. communis* on the bacterial groups was determined separately in the wet, dry or extreme soil by a ratio in two different ways.

1. when the relative abundance of the bacterial group (mean of values obtained after 2, 4 and 6 months) was higher in the uncultivated soil than in the non-rhizosphere or rhizosphere soil, or in the roots then the ratio was calculated as:
  Ratio = (relative abundance of the bacterial group in uncultivated soil – relative abundance of the bacterial group in the non-rhizosphere or rhizosphere soil, or in the roots)/(relative abundance of the bacterial group in the non-rhizosphere or rhizosphere soil, or in the roots).
2. when the relative abundance of the bacterial group (mean of values obtained after 2, 4 and 6 months) was lower in the uncultivated soil compared than in the non-rhizosphere or rhizosphere soil, or in the roots then the ratio was calculated as:

Ratio = −  (relative abundance of the bacterial group in the non-rhizosphere or rhizosphere soil or in the roots − relative abundance of the bacterial group in the uncultivated soil)/(relative abundance of the bacterial group in the uncultivated soil).

## Supplementary Information

Supplementary Information

## Data Availability

The raw sequence data were stored in NCBI Nucleotide Archive under the BioProject: PRJNA550417.
