# Supplementary material for: The root endophytic bacterial community of Ricinus communis L. resembles the seeds community more than the rhizosphere bacteria independent of soil water content
Source: Sci Rep. 2021 Jan 26;11:2173. doi: 10.1038/s41598-021-81551-7 (PMC7838207; doi:10.1038/s41598-021-81551-7)
Supplement: Supplementary file 1 — Supplementary Information [file 41598_2021_81551_MOESM1_ESM.pdf]

Supplementary Material

The root endophytic bacterial community of *Ricinus communis* L. resembles the seeds community more than the rhizosphere bacteria independent of soil water content

Stephanie E Hereira- Pacheco [1], Yendi E Navarro-Noya [2], Luc Dendooven [1*]

[1] Laboratory of Soil Ecology, Cinvestav, Mexico City, Mexico,

[2] Cátedras CONACYT, Centro Tlaxcala de Biología de la Conducta, Universidad Autónoma de Tlaxcala, Tlaxcala, Mexico.

Running title: Bacteria as affected by plant growth in a water stressed soil

***Correspondence:** Luc Dendooven, dendooven@me.com

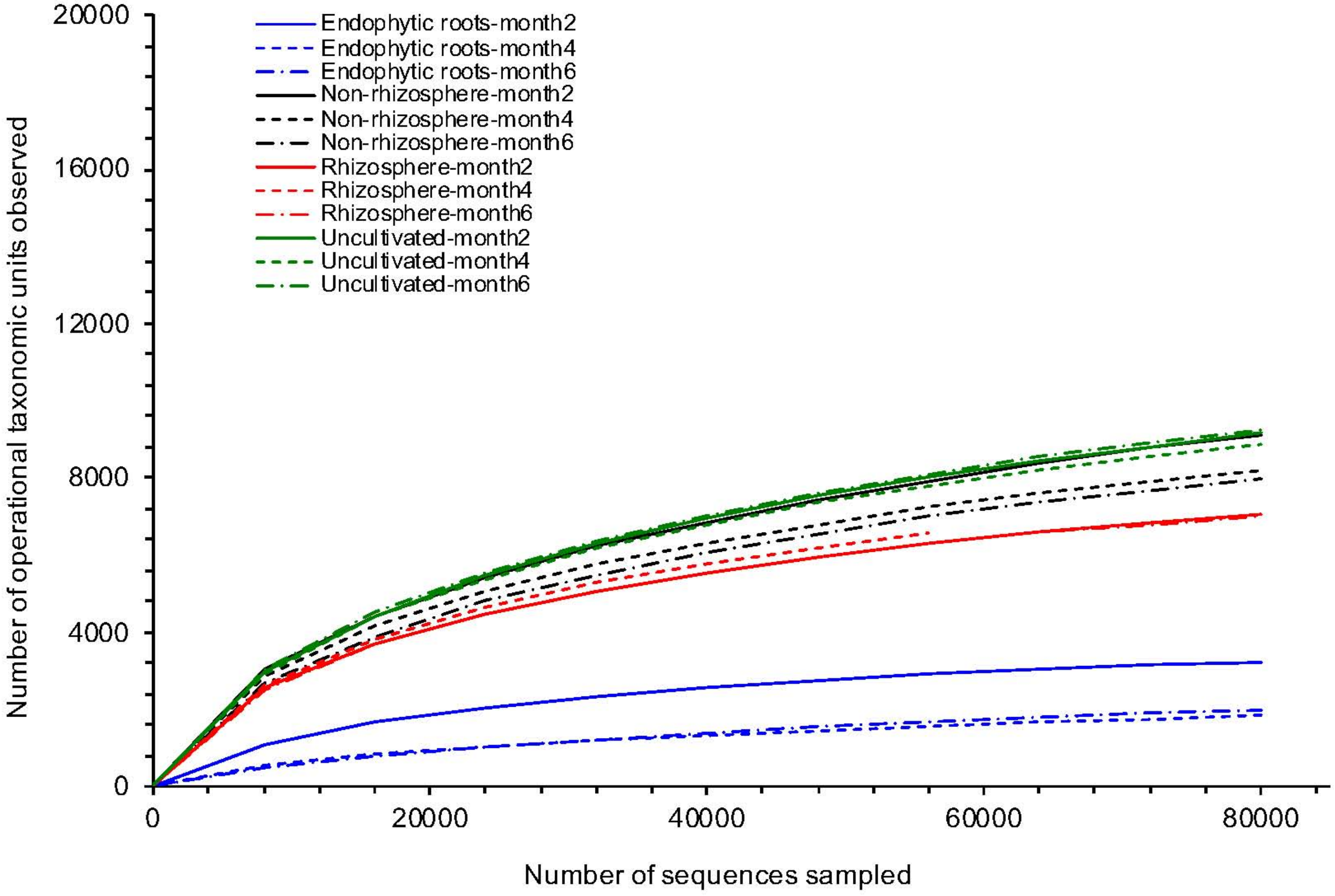

Supplementary Figure S1 Rarefaction curves of the number of sequences analysed versus the number of OTUs obtained in the uncultivated, non-rhizosphere and rhizosphere soil, and the roots after 2, 4 and 6 months.

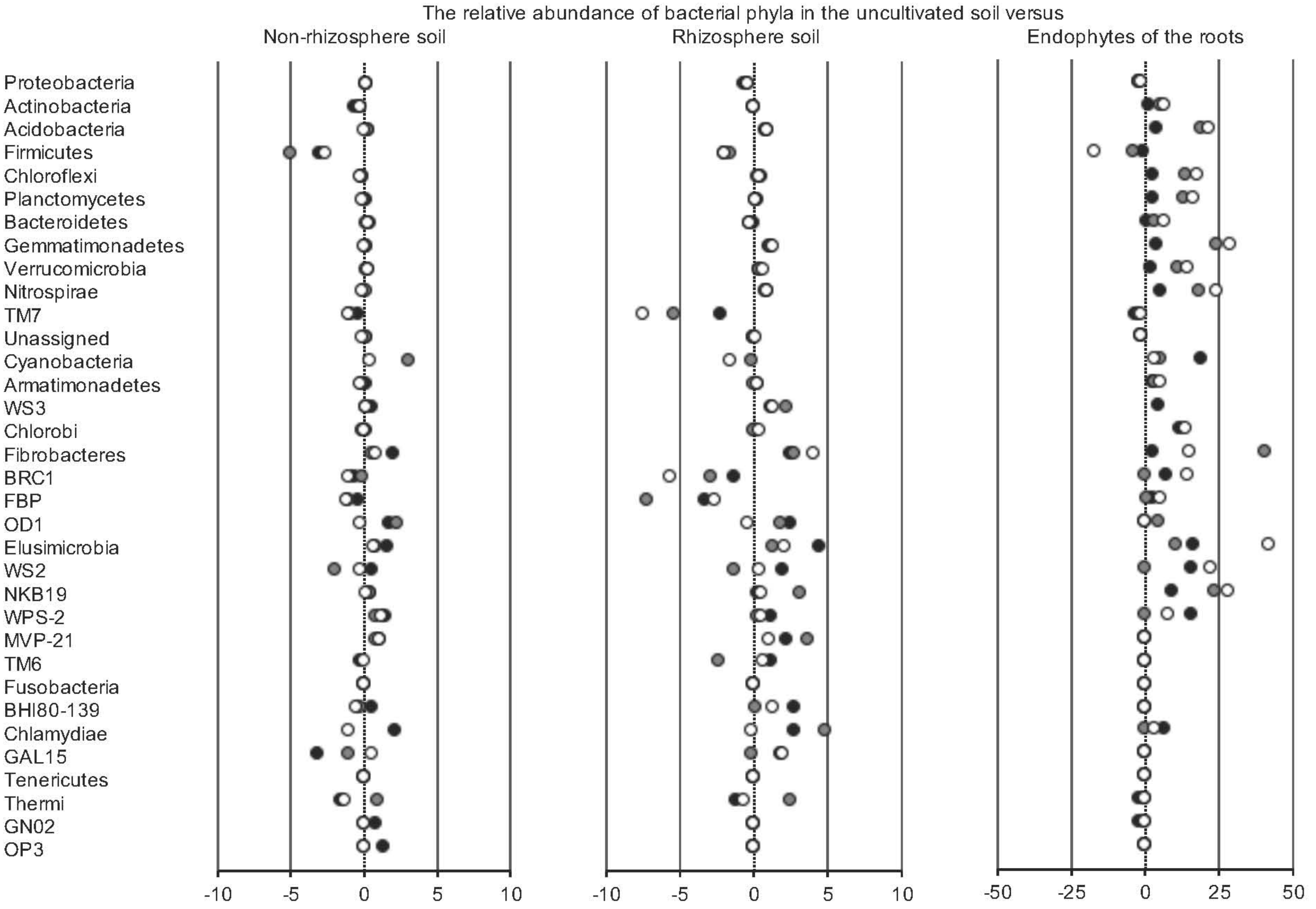

Supplementary Figure S2 Ratio of the relative abundances (mean after 2, 4 and 6 months) of the bacterial phyla in the uncultivated soil versus the non-rhizosphere and rhizosphere soil, and the roots of *Ricinus communis* L in the wet (●), dry (●) and extreme dry soil (○). Wet soil was adjusted to 50% water holding capacity (WHC) with distilled water twice a week, dry soil once every two weeks and extreme dry soil once a month.

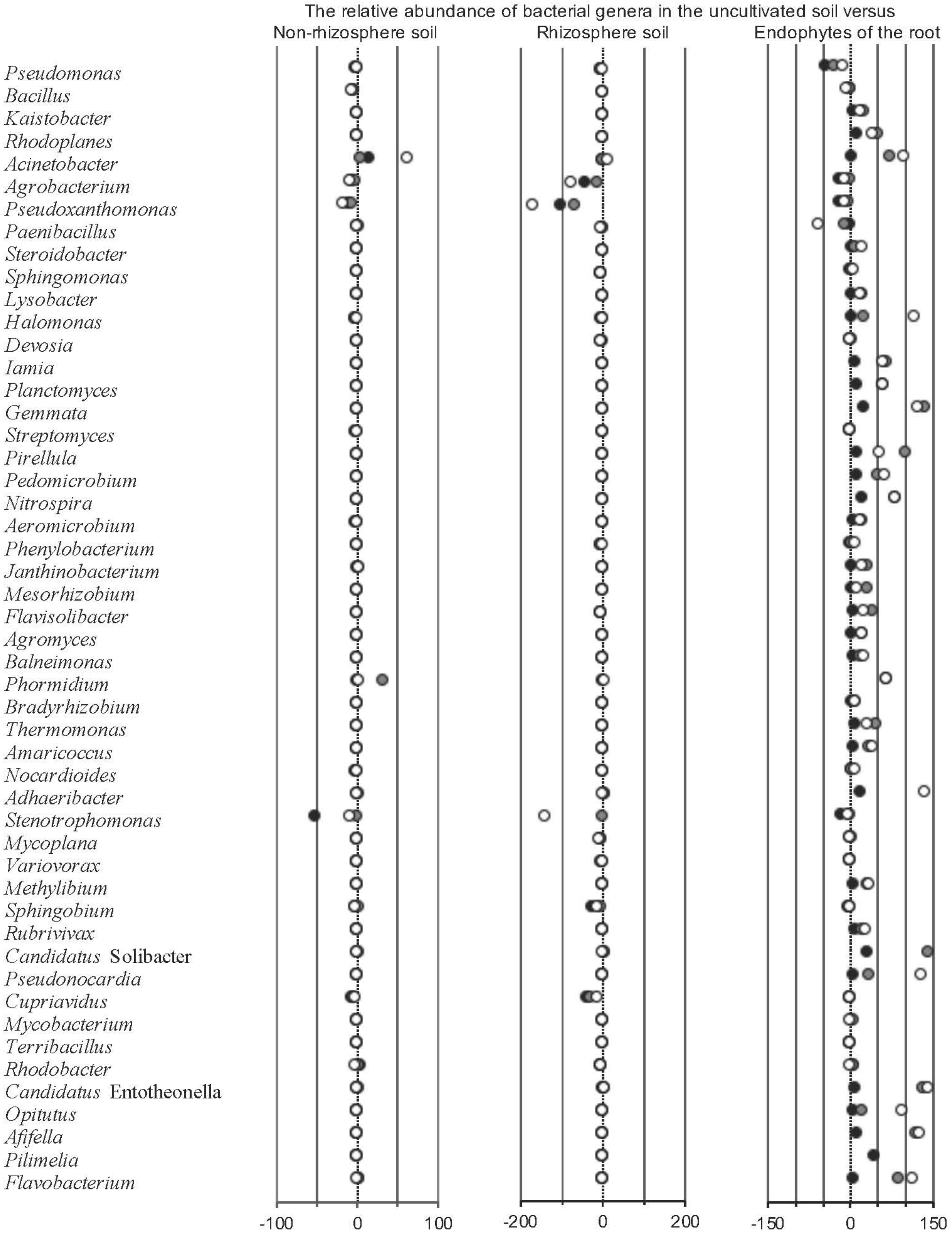

Supplementary Figure S3 Ratio of the relative abundances (mean after 2, 4 and 6 months) of the 50 most abundant bacterial genera in the uncultivated soil versus the non-rhizosphere and rhizosphere soil, and the roots of *Ricinus communis* L in the wet (●), dry (●) and extreme dry soil (○). Wet soil was adjusted to 50% water holding capacity (WHC) with distilled water twice a week, dry soil once every two weeks and extreme dry soil once a month.

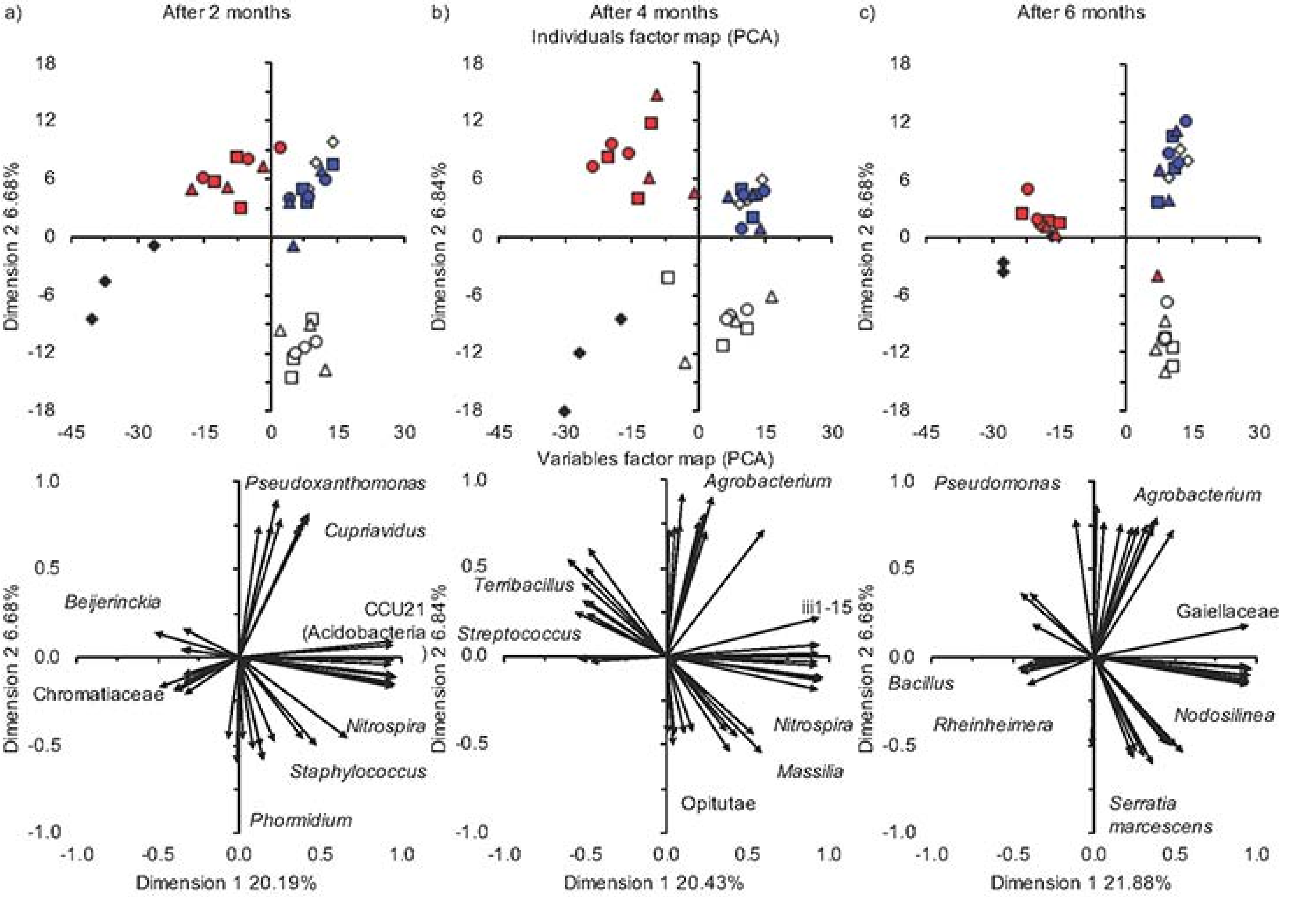

Supplementary Figure S4 A principal component analysis (PCA) with the converted sequence counts of all the bacterial taxa assigned to the level of genus using the centred log-ratio transformation (aldex.clr argument, ALDEx2 package[1]) after a) 2 months, b) 4 months and c) 6 months of applying the different watering regimes in the soil at the onset of the experiment (◇), the rhizosphere in the wet soil (■), the dry soil (●) and the extreme dry soil (▲), the uncultivated wet soil (□), the dry soil (○) and the extreme dry soil (Δ), the endophytic bacteria in the roots of *Ricinus communis* L. in the wet soil (■), the dry soil (●) and the extreme dry soil (▲), and the seeds (◆).Wet soil was adjusted to 50% water holding capacity (WHC) with distilled water twice a week, dry soil once every two weeks and extreme dry soil once a month.

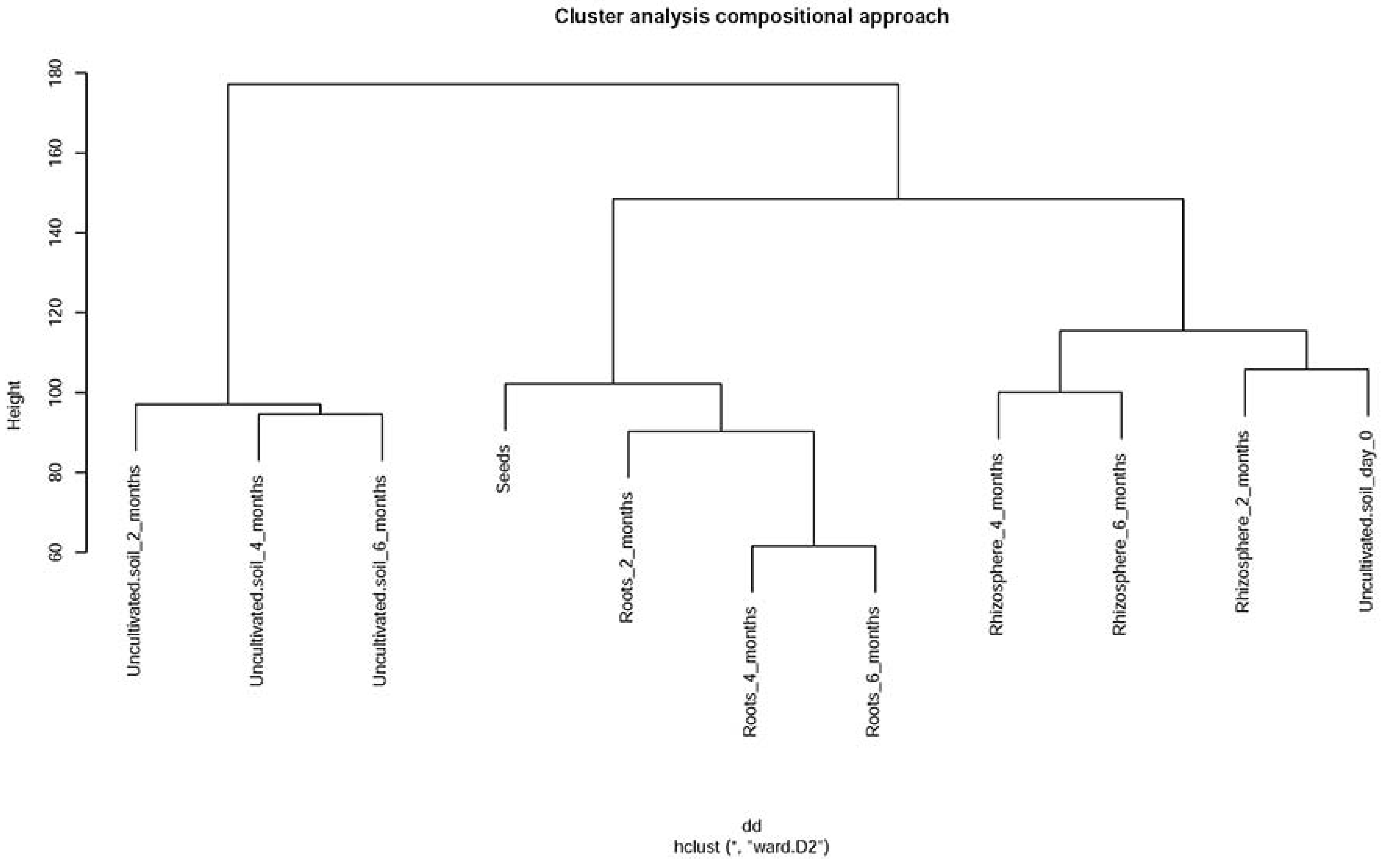

Supplementary Figure S5 Dendrogram of relatedness generated by unsupervised clustering of the Aitchison distances, which is a distance that is robust to perturbations and subcompositions of the data[2] of the uncultivated soil, the rhizosphere soil, the endophytic root bacteria and those found in the seeds.

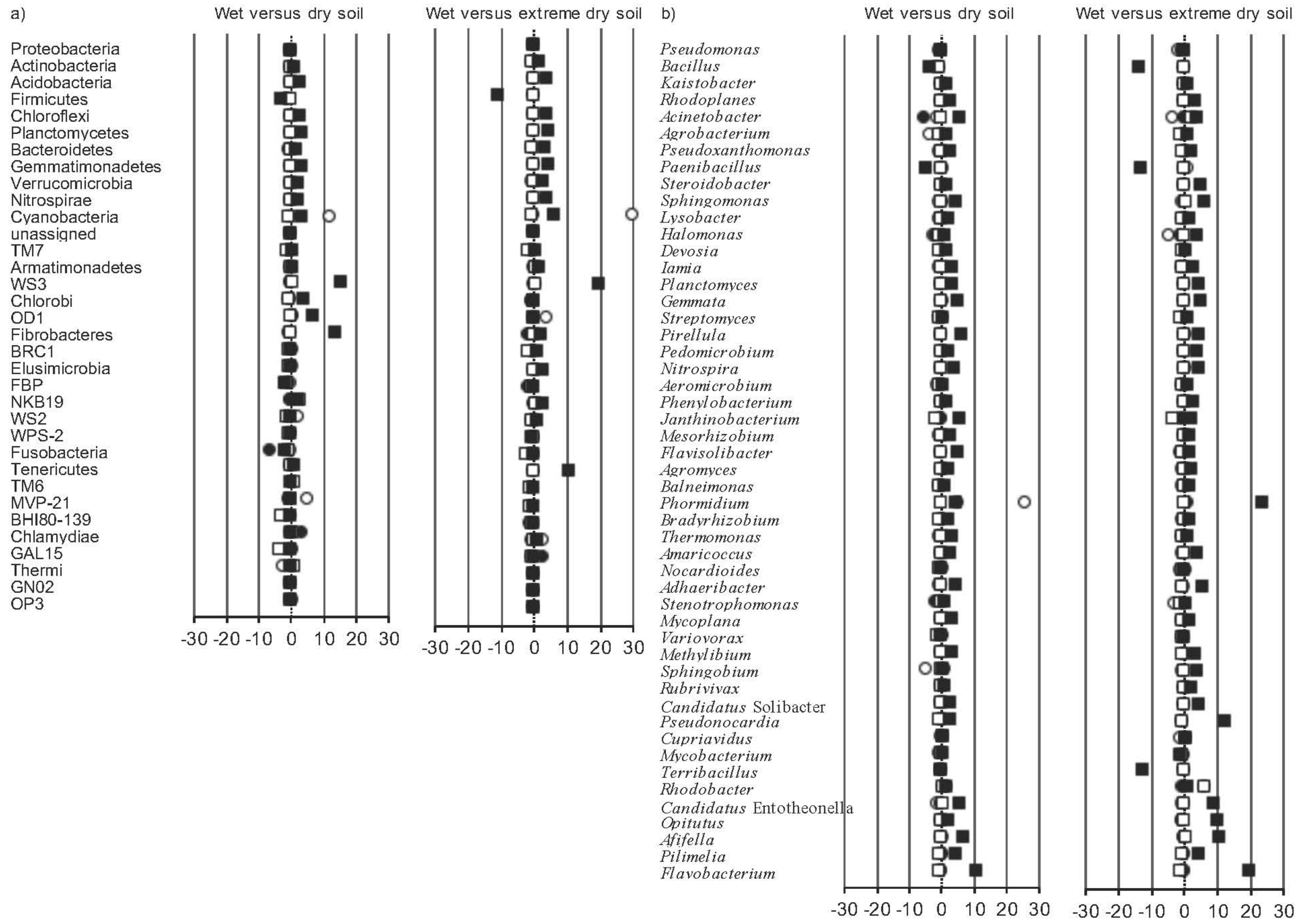

Supplementary Figure S6 Ratio of the relative abundances (mean after 2, 4 and 6 months) of a) the bacterial phyla and b) the 50 most abundant genera in the dry and extreme dry soil versus the wet soil in the uncultivated (○), non-rhizosphere (●) and rhizosphere soil (□), and roots of *Ricinus communis* L (■). Wet soil was adjusted to 50% water holding capacity (WHC) with distilled water twice a week, dry soil once every two weeks and extreme dry soil once a month.

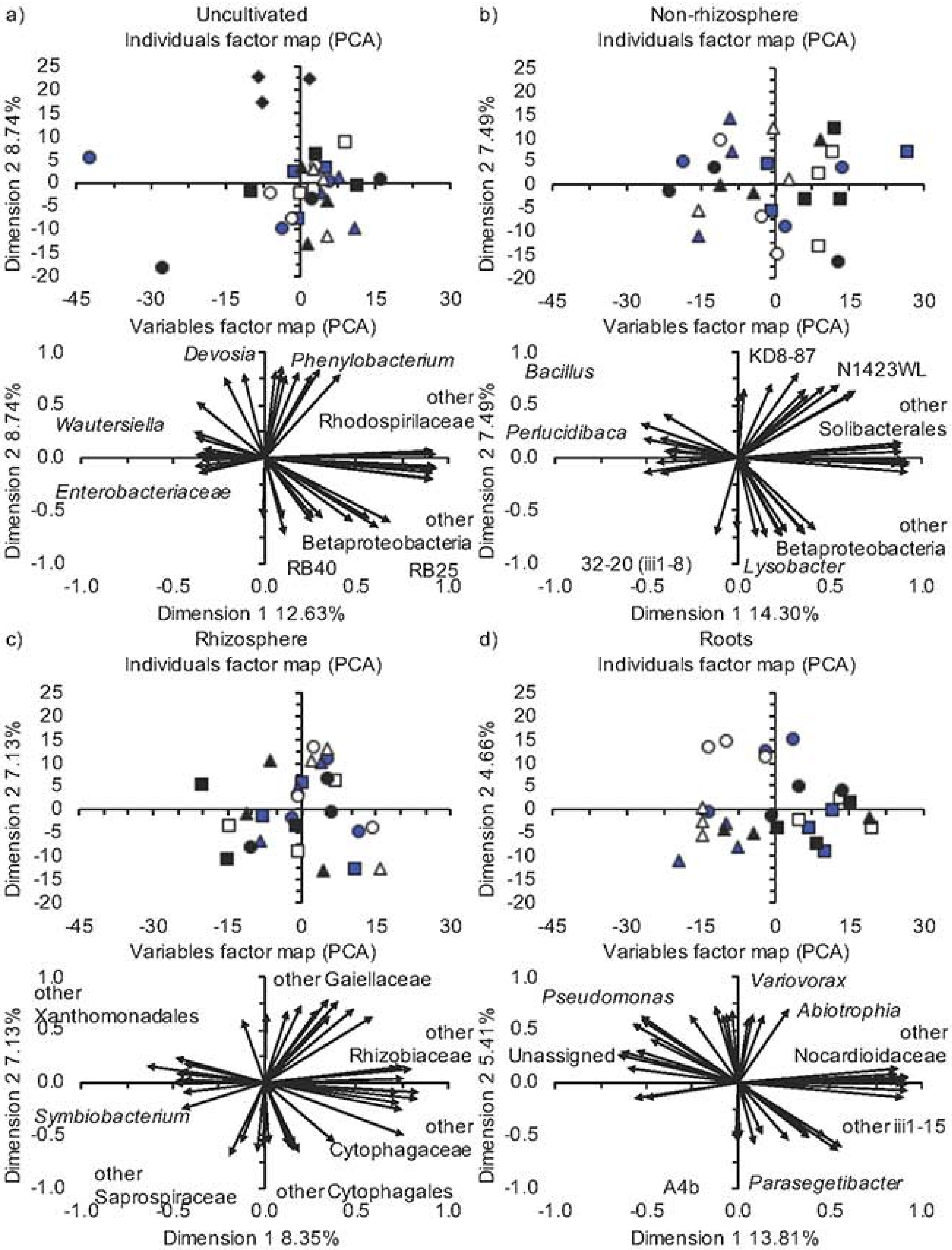

Supplementary Figure S7 A principal component analysis (PCA) with the converted sequence counts of all the bacterial taxa assigned to the level of genus using the centred log-ratio transformation (aldex.clr argument, ALDEx2 package[1]) in in a) the uncultivated, b) the non-rhizosphere, c) the rhizosphere soil, and d) the roots of *Ricinus communis* L. at the onset of the experiment (◆), in the wet soil after 2 (■), 4 (●) and 6 months (▲), dry soil after 2 (■), 4 (●) and 6 months (▲), and the extreme dry soil after 2 (□), 4 (○) and 6 months (△). Wet soil was adjusted to 50% water holding capacity (WHC) with distilled water twice a week, dry soil once every two weeks and extreme dry soil once a month.

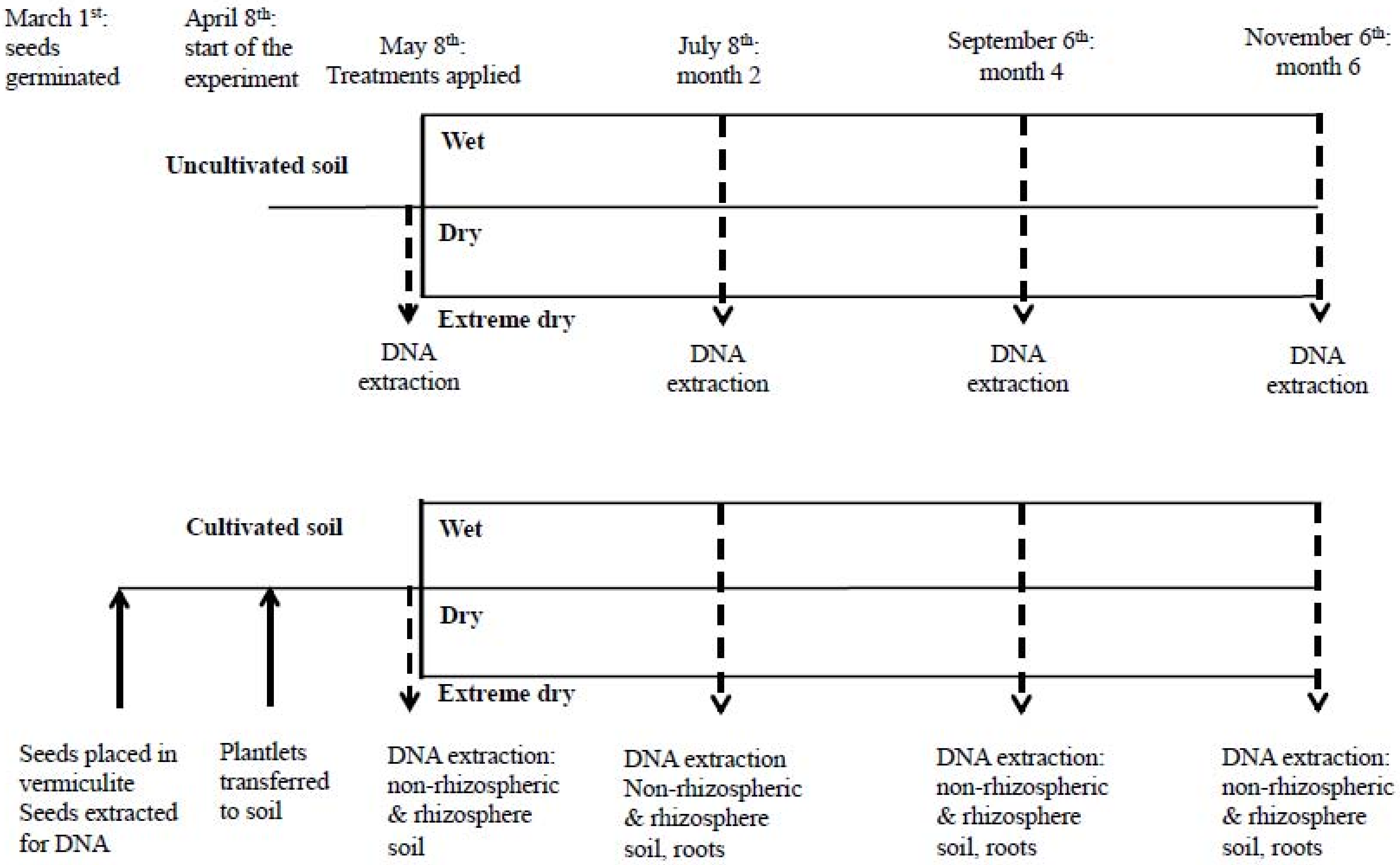

Supplementary Figure S8 Experimental design.

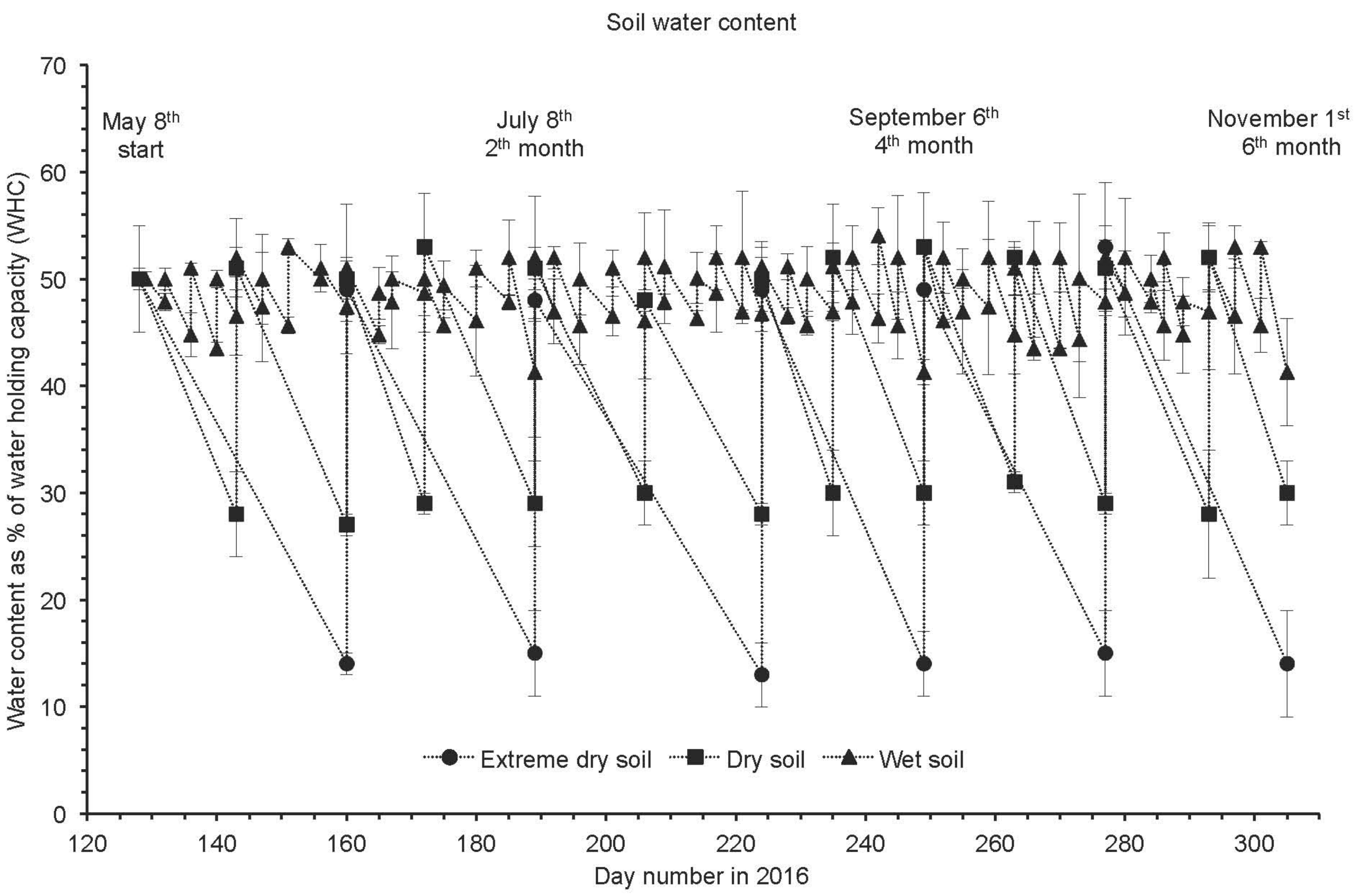

Supplementary Figure S9 Water content in soil during the cultivation of *Ricinus communis* L. for 6 months. The wet soil was adjusted to 50% water holding capacity (WHC) with distilled water twice a week (▲), the dry soil once every two weeks (■) and the extreme dry soil once a month (●).

Supplementary Table S1. Effect of watering regime and time of sampling on the alpha diversity parameters in the uncultivated, non rhizosphere and rhizosphere soil, and roots of cultivation of *Ricinus communis* L.

Effect of watering regime						
Chao1						
Treatment	Uncultivated	Non rhizosphere	Rhizosphere	Roots	F value	*P* value [a]
Wet	4771 a A	4783 a A	4870 a A	1576 a B	61.89	< **0.001**
Dry	4772 a A	4711 a A	4567 a A	1148 a B	107.3	< **0.001**
Extreme dry	4769 a A	4831 a A	4529 a A	940 a B	93.75	< **0.001**
F value	0.08	0.1	2.16	4.85		
P value	0.927	0.906	1.2	0.05		
Simpson index						
Wet	0.162 a A	0.11 a AB	0.094 a AB	0.044 a B	5.15	**0.028**
Dry	0.159 a A	0.122 a A	0.118 a A	0.095 a A	2.43	0.140
Extreme dry	0.138 a A	0.138 a A	0.087 a A	0.053 a A	4.41	**0.041**
F value	0.27	0.76	0.87	2.55		
P value	0.77	0.509	0.468	0.158		
Shannon diversity index						
Wet	10.16 a A	9.97 a A	9.83 a A	5.96 a B	90.9	< **0.001**
Dry	10.09 a A	9.81 a A	9.9 a A	4.97 a B	152.09	< **0.001**
Extreme dry	10.04 a A	10.05 a A	9.68 a A	4.89 a B	104.39	< **0.001**
F value	0.09	0.51	1.1	3.66		
P value	0.918	0.625	0.392	0.091		
Effect of time of sampling						
Chao1						
Month	Uncultivated	Non rhizosphere	Rhizosphere	Roots	F value	p value
2	4815 a A	4875 a A	4661 ab A	2178 a B	52.46	< **0.001**
4	4724 a A	4879 a A	4788 a A	875 b B	14.95	< **0.001**
6	4774 a A	4571 a A	4436 b A	612 b B	97.31	< **0.001**
F value	0.09	0.99	10.81	25.71		
P value	0.915	0.426	**0.01**	< **0.001**		
Simpson index						
2	0.135 a A	0.151 a A	0.091 a A	0.147 a A	1.02	0.434
4	0.128 a A	0.165 a A	0.111 a A	0.026 b B	98.8	< **0.001**
6	0.186 a A	0.055 b BC	0.096 a B	0.019 b C	169.3	< **0.001**
F value	1.49	13.96	0.51	10.35		
P value	0.299	**0.006**	0.622	**0.011**		
Shannon diversity index						
2	10.07 a A	10.18 a A	9.84 a A	8.54 a B	14.96	**0.001**
4	9.93 a A	10.24 a A	9.89 a A	4.38 b B	20.14	< **0.001**
6	10.25 a A	9.4 b A	9.64 a A	2.90 b B	24.18	< **0.001**
F value	0.52	7.14	2.12	55.8		
P value	0.619	**0.026**	0.201	< **0.001**		

[a] *P* values in bold indicate significance.

TABLE S2 Effect of cultivation of *Ricinus communis* L. uncultivated, non-rhizosphere and rhizosphere soil, and roots on bacterial genera and phyla in the wet, dry and extreme dry soil using a compositional approach, i.e. analysis of differential abundance taking sample variation into account ALDEx2 package[1].

**Wet soil**		**Dry soil**		**Extreme dry soil**	
Proteobacteria [a]	1.8×10^{-5} [b]	Gemmatimonadetes	1.2×10^{-5}	Gemmatimonadetes	1.1×10^{-5}
TM7	2.4×10^{-5}	TM7	3.7×10^{-5}	Proteobacteria	3.5×10^{-5}
				TM7	3.6×10^{-6}
Agrobacterium	4.8×10^{-5}	*Candidatus* Entotheonella	1.9×10^{-5}	*Afifella*	1.1×10^{-5}
Candidatus Solibacter	4.3×10^{-6}	*Candidatus* Solibacter	9.2×10^{-6}	*Agrobacterium*	1.6×10^{-5}
Gemmata	7.6×10^{-6}	*Gemmata*	3.2×10^{-5}	*Candidatus* Solibacter	1.1×10^{-5}
Nitrospira	9.7×10^{-6}	*Iamia*	3.5×10^{-5}	*Euzebya*	1.2×10^{-5}
Pedomicrobium	5.8×10^{-5}	*Nitrospira*	2.0×10^{-5}	*Gemmata*	9.4×10^{-6}
Pilimelia	2.3×10^{-5}	*Pedomicrobium*	4.9×10^{-5}	*Nitrospira*	6.4×10^{-6}
Pirellula	3.0×10^{-5}	*Pilimelia*	3.9×10^{-5}	*Pedomicrobium*	4.0×10^{-6}
Planctomyces	4.8×10^{-5}	*Pirellula*	3.7×10^{-5}	*Pilimelia*	1.0×10^{-5}
Pseudomonas	5.4×10^{-5}	*Pseudoxanthomonas*	3.6×10^{-5}	*Pseudonocardia*	4.9×10^{-6}
Rhodoplanes	2.4×10^{-5}	*Rhodoplanes*	1.7×10^{-5}	*Rhodoplanes*	6.1×10^{-6}

[a] Only the bacterial phyla highly significantly and the ten genera most highly significantly affected by location $P < 0.0001$ and with the lowest P values obtained are given, [b] The expected values of the Kruskal-Wallis test for each feature obtained with aldex.kw argument with converted sequence data using the centred log-ratio transform test returned by the aldex.clr argument ALDEx2 package.

TABLE S3 Pairwise PERMANOVA test (corrected *P*-values with the Benjamini-Hochberg adjustment) to determine the effect of cultivation of *Ricinus communis* L. on the bacterial communities (uncultivated soil, non-rhizosphere soil, rhizosphere and roots) considering all OTUs (operational taxonomic units) using a compositional approach [a].

	Wet soil			Dry soil			Extreme dry soil		
	Non-rhizosphere	Rhizosphere	Roots	Non-rhizosphere	Rhizosphere	Roots	Non-rhizosphere	Rhizosphere	Roots
Rhizosphere	0.002			< 0.001			< 0.001		
Roots	< 0.001	< 0.001		< 0.001	< 0.001		< 0.001	< 0.001	
Uncultivated	< 0.001	< 0.001	< 0.001	< 0.001	< 0.001	< 0.001	< 0.001	< 0.001	< 0.001

[a] pairwise perMANOVA analysis with sequence counts converted using the centred log-ratio transform, i.e. aldex.clr argument (ALDEx2 package[1]) (aldex.clr(stephcounts, mc.samples = 1000, denom="all", verbose = FALSE, useMC=FALSE)). The pairwise.perm.manova function from the RVAideMemoire package[3] was used (#pairwise.perm.manova (vegdist(stephclr, "euclidian"), stephcode$cultivation, nperm= 1000, p.method= “BH”).

TABLE S4 Effect of water content (wet, dry and extreme dry) on bacterial groups using a compositional approach, i.e. analysis of differential abundance taking sample variation into account (ALDEx2 package[1]).

**Uncultivated soil**		**Rhizosphere soil**		**Endophytic root**	
Actinobacteria [a]	0.015 [b]	TM7	0.022	Firmicutes	0.030
Cyanobacteria	0.007	*Glycomyces*	0.001	OD1	0.030
OD1	0.022	*Janthinobacterium*	0.018	*Bacillus*	0.033
Verrucomicrobia	0.046	*Rhodobacter*	0.004	*Luteimonas*	0.028
Euzebya	0.026			*Ochrobactrum*	0.046
Hydrogenophaga	0.045			*Promicromonospora*	0.028
Nodosilinea	0.005			*Sphingomonas*	0.017
Phormidium	0.007				
Rhodobacter	0.017				

[a] All bacterial groups that were significantly affected by water content are given ($P < 0.05$), [b] The expected values of the Kruskal-Wallis test for each feature obtained with aldex.kw argument with converted sequence data using the centred log-ratio transform test returned by the aldex.clr argument (ALDEx2 package).

TABLE S5 Effect of water content on the relative abundance of *Actinoplanes, Chromatiales and* Myxococcaceae

Phylum		Actinobacteria	Proteobacteria	
Class		Actinobacteria	Gammaproteobacteria	Deltaproteobacteria
Order		Actinomycetales	Chromatiales	Myxococcales
Family		Micromonosporaceae		Myxococcaceae
Genus		Actinoplanes		
Ratio	Month [a]	Uncultivated soil		
D/W [b]	2	0.97	2.13	0.82
E/W [c]	2	1.09	0.52	0.96
D/W	4	1.86	1.65	2.55
E/W	4	1.43	1.59	1.92
D/W	6	0.90	1.12	0.77
E/W	6	1.55	1.23	1.44
		Non-rhizospheric soil		
D/W	2	1.70	0.69	1.16
E/W	2	1.32	0.59	0.77
D/W	4	0.71	0.61	0.87
E/W	4	1.09	0.53	1.34
D/W	6	1.50	0.32	1.07
E/W	6	2.30	0.35	1.38
		Rhizosphere		
D/W	2	3.27	1.30	2.61
E/W	2	7.20	0.00	1.53
D/W	4	1.77	0.78	1.11
E/W	4	3.45	0.36	1.06
D/W	6	0.83	2.27	0.85
E/W	6	0.48	0.31	0.80
		Roots		
D/W	2	0.69	ND	0.72
E/W	2	0.66	ND	0.38
D/W	4	0.60	ND	0.49
E/W	4	0.90	ND	0.00
D/W	6	1.81	ND	0.00
E/W	6	3.30	ND	0.00

[a] Time elapsed since the different water regimes (i.e., wet, dry and extreme dry) were applied to soil, [b] Ratio of the relative abundance of the bacterial group in the dry soil versus the wet soil (D/W), [c] Ratio of the relative abundance of the bacterial group in the extreme dry soil versus the wet soil (E/W).

References to the Supplementary Material

1. Gloor, G et al. ALDEx2 package: Analysis of differential abundance taking sample variation into account. Version: 1.18.0 Date: 2019-10-25 (2019).
2. Aitchison, J. The statistical analysis of compositional data. Chapman and Hall, London, UK. (1986).
3. Hervé M. Testing and plotting procedures for biostatistics. In: Package 'RVAideMemoire'; 2018.
